# Pathogenicity of Misfolded and Dimeric HLA-B27 Molecules

**DOI:** 10.1155/2011/486856

**Published:** 2011-03-30

**Authors:** Antony N. Antoniou, Izabela Lenart, David B. Guiliano

**Affiliations:** Division of Infection and Immunity/Centre of Rheumatology, Department of Immunology and Molecular Pathology, University College London, Windeyer Institute of Medical Science, 46 Cleveland Street, London W1T 4JF, UK

## Abstract

The association between HLA-B27 and the group of autoimmune inflammatory arthritic diseases, the spondyloarthropathies (SpAs) which include ankylosing spondylitis (AS) and Reactive Arthritis (ReA), has been well established and remains the strongest association between any HLA molecule and autoimmune disease. The mechanism behind this striking association remains elusive; however animal model and biochemical data suggest that HLA-B27 misfolding may be key to understanding its association with the SpAs. Recent investigations have focused on the unusual biochemical structures of HLA-B27 and their potential role in SpA pathogenesis. Here we discuss how these unusual biochemical structures may participate in cellular events leading to chronic inflammation and thus disease progression.

## 1. Introduction

The association between HLA-B27 and the group of autoimmune inflammatory arthritic conditions referred to collectively as the Spondyloarthropathies (SpAs) is well established and has been known for over 30 years [1, 2]. Of all the SpAs, Ankylosing Spondylitis (AS) exhibits the strongest association with HLA-B27. Within the UK, the current Arthritis Research UK estimates for the number of AS cases are between 116–318,000 (male) and 29–87,000 (female) (http://www.arthritisresearchuk.org/research/data_on_arthritis/data_on_as.aspx) of which 95% are HLA-B27^+^. Reactive arthritis (ReA), an SpA where joint inflammation develops following certain bacterial infections, such as *Salmonella* and *Chlamydia*, has an association with HLA-B27 up to 80% [3]. The SpAs are multisystemic in nature, and the major targets of the disease are axial and peripheral joints (which in its severest form can lead to bone fusion of the sacroiliac joint), entheses (points of ligament, tendon and joint capsule insertion into bone), skin (psoriasis or psoriasis-like lesions), mucosa, gut (inflammatory bowel disease), urinogenital tract, and eyes.

## 2. HLA-B27 and the Spondyloarthropathies

The Human Leukocyte Antigen (HLA)-B27 is a Major Histocompatibility Complex (MHC) class I molecule encoded within the HLA-B locus. MHC class I molecules present peptides, derived from intracellular antigens, to CD8^+^ cytotoxic T cells, and act as ligands for Natural Killer cells. Like other MHC class I molecules it is a glycoprotein that folds within the lumen of the endoplasmic environment (ER). It is composed of a heavy chain noncovalently associated with the light chain beta-2-microglobulin (*β*2m) and loaded with a 8–13 amino acid long peptide before expression at the cell surface [4]. 

The most significant observation implicating the direct involvement of HLA-B27 in SpAs has come from HLA-B27 transgenic rats. Several HLA-B27-human *β*2m rat transgenic lines developed a spontaneous multisystemic inflammatory arthritic disorder with many features of human AS [5, 6]. However, the rat transgenic models do exhibit subtle differences to human AS, such as the order of appearance and frequency of certain disease features. Transmission of disease has remained stable through the generations, and most backgrounds tested, Lewis, Fisher, and PVG, are permissive whilst the Dark agouti strain is resistant. Therefore, like the human disease, other susceptibility genes are probably involved [6, 7].

There are two main theories which attempt to explain the link between HLA-B27 and the SpAs. The first is the “arthritogenic peptide hypothesis” which postulates that specific pathogenic peptide(s) derived from the target organ are presented to self-reactive T cells [8]. A variation on this theme of antigen processing is “molecular mimicry” where a cross-reactive peptide derived from an infecting bacterial pathogen stimulates T cells that can respond to an HLA-B27 associated “self-peptide” or to peptides derived from HLA-B27 itself [8]. The second hypothesis proposes that certain biochemical characteristics of HLA-B27, mainly its ability to misfold, were the main contributory factor leading to SpA development [9–11]. This latter idea is referred to as the “misfolding hypothesis” and is the main focus of this paper.

## 3. The Misfolding Hypothesis

The misfolding hypothesis postulates that HLA-B27 can misfold within the environment of the ER and lead to a “gain of toxicity function” by inducing cellular stress responses. These responses are known as the unfolded protein response (UPR) and the ER overload response (EOR). This in turn would perturb normal cellular function and lead to proinflammatory cytokine production contributing to SpA development [10]. The misfolding hypothesis has led to the interesting notion that the SpAs could be reclassified as autoinflammatory rather than autoimmune in nature. Such autoinflammatory diseases are characterized by inflammation in the absence of autoantibodies and antigen specific-T cells, which are two criteria that the SpAs seemingly fullfill [12]. 

**Figure 1:**
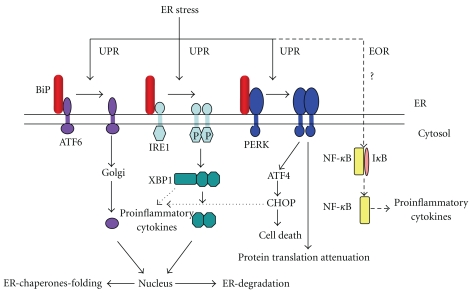
Schematic illustrating the basic outline of the ER stress pathways. The effector molecules of the UPR, the transcription factor ATF6, and the kinases IRE1 and PERK are held in an inactive state via their association with the ER chaperone BiP. On sensing misfolding, BiP dissociates from these effector molecules. This results in ATF6 trafficking to the Golgi where two Golgi resident proteases cleave the ATF6 cytosolic domain which acts as a transcription factor activating folding chaperones. IRE1 oligomerises and autophosphorylates, which can splice the 26-base pair intronic sequence from XBP1 mRNA, which is then religated and encodes for a potent transcription factor. XBP1 induces the production of chaperones and proteins involved in protein folding or degradation. PERK oligomerises and autophosphorylates which in turn phosphorylates the elongation factor 2*α* that inhibits protein translation. PERK can activate ATF4 and CHOP which can activate apoptosis. Since being reported, little is known about the EOR, which is thought to detect a build-up of proteins within the ER and activate NF-*κ*B, which can target proinflammatory cytokine production.

The origins of the misfolding hypothesis stemmed from a key biological observation made in the mid-1990s. In yeast and later in mammalian cells, it was shown that by disrupting global protein folding within the ER, cellular events trigger activation of genes and production of proteins whose functions aim at restoring ER homeostasis [13, 14]. These cellular stress responses are referred to as ER stress responses, of which there are two types, the Unfolded Protein Response (UPR) and ER Overload Response (EOR) (Figure 1). The UPR aims to restore ER homeostasis whilst dealing with misfolded proteins, while the EOR attempts to restore normal ER function during the handling of accumulating protein. 

There are three main effector molecules of the UPR, the ATF6 transcription factor and the two kinases IRE1 and PERK [14–16]. These effector molecules reside within the ER and are maintained in an inactive state by the ER resident chaperone Immunoglobulin Binding Protein (BiP). During ER stress, BiP is sequestered away from these effector molecules, leading to their activation [17–19] (Figure 1). 

Dissociation from BiP unmasks targeting signals, which translocate ATF6 to the Golgi apparatus. Within the Golgi apparatus, proteolytic processing of the ATF6 cytosolic domain by two resident proteases SP1 and 2 occurs [17, 20]. The ATF6 cytosolic domain is then translocated to the nucleus and acts as a potent transcription factor upregulating chaperones participating in protein folding [20, 21]. Prolonged ER stress can induce the oligomerisation and autophosphorylation of the ER resident IRE1 and PERK kinases [22]. Phosphorylated IRE1 can splice the cytosolically located XBP1 mRNA by removing a 26-nucleotide intronic sequence which then generates the potent XBP-1 spliced(s) transcription factor [23]. XBP1s can activate chaperones to enhance cellular folding capacity and/or proteins involved in degradation of misfolding substrates [24]. Activated PERK can phosphorylate elongation factor 2*α* (eF2*α*), which inhibits protein synthesis, thus reducing the protein load within the ER [25, 26]. Furthermore PERK can activate a series of transcription factors (ATF4 and CHOP) which can lead to the induction of apoptosis, thus leading to the removal of terminally distressed cells [27–30]. 

The ER overload response remains poorly defined, but it is thought that accumulation of misfolded proteins within the ER leads to the activation of the transcription factor NF-*κ*B [31, 32] (Figure 1). As NF-*κ*B can target proinflammatory cytokine genes, activation of this transcription factor could be a key link between HLA-B27 misfolding and the production of proinflammatory cytokines. However, since the proposition of these ER stress pathways, in stark contrast to the UPR, which has been well characterized, the EOR remains ill defined. 

Perhaps the most significant finding demonstrating a link between elevated ER stress induction and the production of proinflammatory mediators and cytokines came from the demonstration that ER stress within the liver led to the activation of the transcription factor, CREBH, which is a member of the same family of b-ZiP transcription factors to which ATF6 belongs. CREBH activation directly led to enhanced production of proinflammatory mediators [33]. Subsequently, other transcription factors of the ER stress pathway can be involved in proinflammatory cytokine production such as XBP-1 [34] and CHOP [35]. Furthermore, proinflammatory cytokines, notably TNF*α*, can induce ER stress [36, 37]. This raises the possibility that, if HLA-B27 misfolding does induce ER stress and lead to proinflammatory cytokine production, this could develop into a positive feedback loop which exacerbates and perpetuates the release of TNF*α* and other pro-inflammatory mediators. Thus there does indeed appear to be a link between ER stress and a proinflammatory cytokine environment, which is a characteristic feature of AS patients.

## 4. The Misfolding Hypothesis and HLA-B27 Rat Transgenic Models

Certain features of the rat model suggest that antigen presentation by HLA-B27 alone cannot be responsible for disease. Firstly, there is a strict correlation between susceptibility to disease and levels of HLA-B27 as determined by transgene copy number. A threshold of HLA-B27 copy number exists above which disease predictably arises [38]. However, such copy number dependency calls into question whether disease in rats was specific or merely a toxic side effect of transgene overexpression. Disease development was HLA-B27 specific as HLA-B7 rats with an identical copy number failed to develop any pathology [6]. Intriguingly, one human study demonstrated that elevated levels of HLA-B27 within AS patients could be a determining factor in disease development [39]. Further studies established that some features of the rat SpAs could be transferred to both HLA-B27 disease resistant and nontransgenic recipients by bone marrow engraftment of immature hematopoietic cells [40]. Of the disease features, it was the associated colitis and psoriasiform skin lesions that were predominantly transferred by bone marrow engraftment [40]. Thymic exposure to HLA-B27 was therefore not necessary but T cells were required for disease induction [41]. Somewhat surprisingly though, T cells from HLA-B27 transgenic rats of disease prone lines were not able or necessary to induce disease in irradiated nontransgenic recipients [41]. Further evidence arguing against a traditional role for cytotoxic T cell recogniton of HLA-B27 was the demonstration that CD4^+^ T cells were more efficient than CD8^+^ T cells in transferring disease and that CD8^+^ T cells displayed a nonactivated profile in disease-prone strains [42]. A distinct lack of CD8^+^ T cell involvement in AS was further confirmed by the recent studies in CD8 knockouts in the HLA-B27 rat transgenics [43]. 

Bone marrow-derived macrophages (BMDMs) derived from HLA-B27-transgenic rats with inflammatory arthritis were demonstrated to exhibit cellular stress responses in the form of the UPR expressing elevated levels of XBP-1s [44]. However, the generation of high copy number HLA-B27. human *β*2m rat transgenic model, designed to “improve the folding” of HLA-B27 did not alleviate, but instead, exacerbated skeletal disease. This transgenic rat did not develop the colitis that is a common feature of the rat “AS-like” disease suggesting that HLA-B27 misfolding could participate in distinct forms or facets of inflammatory arthritic disease [45]. However, analysis of transgenic animals under conditions enhancing human *β*2m expression as well as HLA-B27 heavy chain did demonstrate UPR activation [46]. 

A major conundrum in the generation of AS animal models is that several HLA-B27 transgenic mouse strains remain healthy. However, this could simply reflect the genetic backgrounds of the strains that were chosen for these studies. Some mouse strains do develop a form of inflammatory arthritis that has been linked to MHC class I molecules. ANKENT, a spontaneous and progressive ankylosing enthesopathy affecting ankle/tarsal joints of ageing mice, demonstrates both MHC and non-MHC linked predisposition, male predominance, and histological enthesitis. The mouse H-2^*k*^ haplotype appears to be an important risk factor, and the introduction of HLA-B*2702 increased the incidence of joint tarsal ankylosis [47–49]. Also, disrupting the MHC class I pathway either by knocking out the light chain *β*2m or TAP (which actively transports peptides into the ER lumen for MHC class I presentation) can lead to the spontaneous development of a mild arthritis on some mouse genetic backgrounds. These latter observations were made independent of HLA-B27, but support the idea that misfolding of MHC class I molecules can lead to a proinflammatory arthritic phenotype [50].

## 5. The Misfolding Hypothesis and Biochemical Characteristics of HLA-B27

Biochemical analysis over the last 10 years has demonstrated that HLA-B27 displays several characteristics that, to date, set it apart from most other MHC class I molecules. The relationship between ER stress pathways and HLA-B27 was postulated following biochemical observations demonstrating that HLA-B27 exhibited an enhanced tendency to misfold [9] and was susceptible to aggregation [51]. 

A combination of residues in the B pocket (which binds the N-terminal motif of MHC class I associated peptides) were initially recognised as characteristic to HLA-B27 when compared to other B alleles. These included the residues, His9, Thr24, Glu45, Cys67, Lys70, Ala71, and Gln97, which line the B pocket and accommodate the second anchor residue of associated peptides. This observation was thought to support the idea that HLA-B27-specific arthritogenic peptides could be presented. However, expressing a combination of these residues to other MHC class I molecules resulted in enhanced misfolding of non-SpA-associated HLA molecules [9, 52]. 

**Figure 2:**
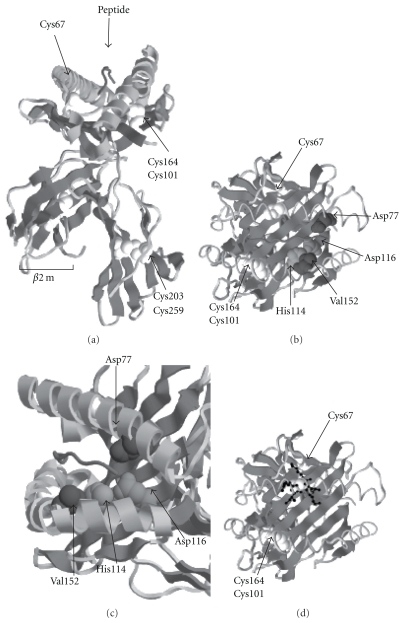
HLA-B27 structure. (a) Ribbon structure of HLA-B27 heavy chain in association with *β*2m and peptide. Structurally conserved cysteines at positions 101, 164, 203, and 258 and the unpaired cysteine at p67 are highlighted by Van der Waals radii. (b) Overhead view of the HLA-B27 antigen binding groove highlighting Cys67 and residues (His114, Asp116, Asp77, and Val152) which are polymorphic between the disease-associated HLA-B*2705 and nondisease-associated HLA-B*2706 subtypes. (c) Head on view of the F pocket of the HLA-B27 antigen binding groove. His114 and Asp116 make up the floor of the F pocket, whilst Val152 and Asp77 can contribute to the interaction between HLA-B27 and the carboxy-terminus of the associated peptide. (d) Overhead view of the HLA-B27 antigen binding groove, with Cys67 (Van der Waals radii) and surrounding residues (ball and stick) are depicted, which are unique to HLA-B27 and contribute to the B pocket of HLA-B27.

If SpA predisposition is related to molecular structure, then specific residues must be involved that differ between the AS predisposing and the HLA-B27 subtypes not associated with AS. Two such HLA-B27 subtypes, HLA-B*2706 and 09, exhibit a weak or no association with the SpAs, but these predominantly differ at residues constituting the F pocket at position (p) 114–116, which bind the peptide anchor motif at p9 [53] (Figures 2(b) and 2(c)). These structural differences would suggest that peptide presentation may play a significant role in SpA development. However, peptide elution and sequencing analysis have revealed that there is significant overlap in the peptides bound by HLA-B*2709 and 06 and disease-associated subtypes [54]. Biochemical analysis does reveal that these subtypes exhibit significant differences in their folding and misfolding characteristics [55–57]. It must be noted that the capacity of HLA-B27 subtypes to form dimers and their relationship to the misfolding hypothesis has yet to be fully tested. The recent genetic association between the Endoplasmic Reticulum Amino Peptidase (ERAP1) [7, 58], which participates in the generation of MHC class I associated peptides, may support a role for the peptide repertoire in determining the misfolding status of HLA-B27. 

In addition to the enhanced propensity to misfold, it was observed that HLA-B27 formed aggregates when purified as a recombinant protein [51]. This observation was later confirmed *in vivo* by several laboratories, which detected both ER resident and cell surface dimers [52, 59–61] (Figure 4). HLA-B27 dimers were demonstrated to correlate with disease incidence in the HLA-B27 transgenic rat models, thus suggesting that these aggregate structures could well indeed be responsible for the “toxicity gain of function” [62]. Intriguingly, HLA-B27 dimeric structures that form within the ER, do not traffic through the secretory pathway, whilst cell surface dimers appear to arise from recycling HLA-B27 molecules [59, 61] (Figure 4). Originally, HLA-B27 dimerisation was proposed to occur through an unpaired cysteine at p67, which forms part of the B pocket of the peptide binding groove (Figures 2(a) and 2(d)) [51]. It appears that Cys67 has a more significant role in dimerisation at the cell surface, whilst those within the ER can form through the structurally conserved cysteine at p164 [52, 59] (Figure 4). Transgenic rats expressing a mutant HLA-B27 molecule lacking Cys67 continue to develop disease but with a lower prevalence [6]. Thus, whether Cys67 plays a significant role in dimer formation and whether HLA-B27 dimers can be used as markers for SpA development remain unresolved. 

HLA-B27 heavy chain dimerisation within the ER appears to be influenced by the maturation kinetics of HLA-B27 [52] and can be induced following differentiation of the dendritic-like cell line KG1.1 [63]. These observations suggested that the slow maturation rate of HLA-B27 allows for inappropriate disulfide linkages to be made. These aggregates can form under physiological conditions, in cell types thought to participate in disease development and under some conditions such as those where MHC class I expression is upregulated. Other HLA-B27 dimeric structures have been reported, which depend on the cytoplasmic-located cys325 [64]. 

Thus the biochemical observations, outlined above, and much of the data relating to animal models of inflammatory arthritis suggest that the biosynthesis of HLA-B27 plays a significant role in disease development.

## 6. Is Pathogenicity Related to HLA-B27 Misfolded Monomeric and/or Dimeric Molecules?

**Figure 3:**
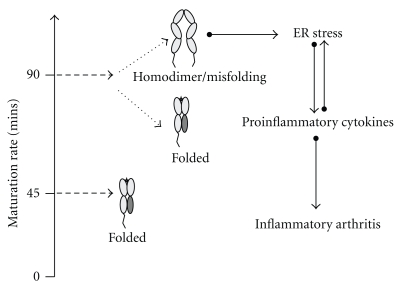
Schematic outline of a hypothetical model of HLA-B27-induced inflammatory arthritis by misfolding and/or dimerisation. HLA-B27 matures slowly resulting in enhanced formation of aggregate structures, which can potentially induce ER stress responses and activate proinflammatory cytokine production. ER stress can be perpetuated by further proinflammatory cytokine production, and this can lead to the development of inflammatory arthritis.

**Figure 4:**
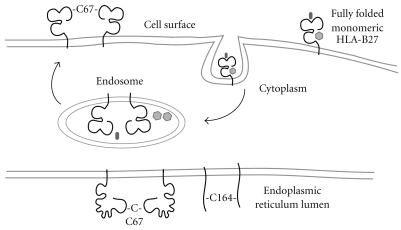
Schematic illustrating that ER resident and cell surface dimers are not related. ER resident dimers can form via C67-C67 or C164-C164 disulfide bonds, but do not transit out off the ER. Cell surface dimers are thought to form following the recycling of fully folded HLA-B27 cell surface molecules through the endocytic pathway before being reexpressed as dimers mediated by C67-C67 interactions.

Recently, certain disorders have been categorised as protein misfolding/conformational diseases, which are characterised by both the loss of function associated with specific protein mutations and “gain of toxicity function” as a result of inappropriate folding. Human disorders attributed to the loss of function of proteins due to mutations, such as cystic fibrosis, or additional gain of toxic function attributed to misfolding and/or aggregation such as neurodegenerative disorders and amyloidosis, have been categorised as conformational diseases [65]. HLA-B27 possesses the two characteristics shared by proteins implicated in conformational diseases, that is, the ability to misfold and aggregate into dimers (Figure 3).

It remains undetermined whether HLA-B27 heavy chain misfolding, the formation of dimers, or both are responsible for ER stress induction and/or participate in AS pathogenesis. The predisposition of HLA-B27 heavy chain to misfolding can be viewed as the precursor to dimerisation. 

HLA-B*2705 heavy chain has been demonstrated to exist in several conformations *in vivo* [66] with each form exhibiting different redox states [66, 67]. A comparison of disease-associated and nonassociated subtypes indicated that HLA-B*2706 and 09 subtype heavy chains were less susceptible to misfolding on the basis of their accessibility to thiol modifying reagents [66]. MHC class I molecule dimerisation is not unique to HLA-B27 and has been reported for other HLA alleles [68–70]. Other HLA molecules can dimerise either under slow folding conditions or when overexpressed ([52] and our unpublished data). Thus, this raises the question, if other HLA class I molecules can dimerise, why are HLA-B27 dimers detectable at steady state? It is possible that HLA-B27 disulfide bonded heavy chain-dimeric structures could be less susceptible to degradation. Such resistance to degradation could reflect complex conformations attained due to both structurally conserved cysteines and the unpaired cysteines at p67, 308, and 325. Misfolded MHC class I heavy chains within the ER are degraded by the proteasome, by a process referred to as ER-associated degradation (ERAD) which involves misfolding protein removal from the ER to the cytosol [71]. Studies analyzing the immunomodulatory effects of human cytomegalovirus (HCMV) gene products, US2 and US11 [72], suggest that MHC class I molecules can use two distinct ERAD pathways. The first involves a degradation complex composed of Derlin 1, the AAA ATPase p97, the E3 ubiquitin ligase, HRD1 and adaptor protein SEL1 [73]. The second pathway is less well characterised and uses the signal peptide peptidase and TRC8 E3 ubiquitin ligase [74, 75]. It would appear that HLA-B27 does not form a single distinct dimeric structure but can form different dimers, probably differing in their conformations [52, 61, 76]. These aggregate structures could well be resistant or inefficiently disposed off by the degradation machinery used, thus their prolonged half life could potentially lead to these structures being pathogenic in a similar but not identical mechanisms proposed for conformational diseases.

## 7. Summary

The misfolding hypothesis, which attempts to explain the association between HLA-B27 and AS, incorporates many of the observations made both with transgenic animal models and at the molecular biochemical level. It is possible that certain facets of the SpAs, for instance, gut inflammation, are more dependent on HLA-B27 misfolding. However, it remains to be determined why certain individuals are predisposed to AS and why certain biological sites such as the entheses are the major targets for SpA development.
